# Supplementary material for: Cerebral topographies of perceived and felt emotions
Source: Imaging Neurosci (Camb). 2025 Mar 27;3:imag_a_00517. doi: 10.1162/imag_a_00517 (PMC12319791; doi:10.1162/imag_a_00517)
Supplement: Supplementary Material [file imag_a_00517-supp.pdf]

Supplementary Information

Cerebral Topographies of Perceived and Felt Emotions

Saarimäki H, Nummenmaa L, Volynets S, Santavirta S, Aksiuto A, Sams M, Jääskeläinen IP, Lahnakoski JM

Correspondence to: heini.saarimaki@tuni.fi or j.lahnakoski@fz-juelich.de

Contents

Supplementary Note 1 2
Supplementary Figure S1. 3
Supplementary Figure S2. 4
Supplementary Figure S3. 5
Supplementary Figure S4. 6
Supplementary Figure S5. 7
Supplementary Table S1. 8
Supplementary Table S2. 11
Supplementary Table S3. 13
Supplementary Table S4. 15
Supplementary Table S5. 15
Supplementary Table S6. 15
Supplementary Table S7. 15
Task instructions 16
- Original Finnish welcome text 16
- English translation of welcome text 17
- Original Finnish instructions to the rating tool 18
- English translation of instructions 20

Supplementary Note 1

To enable parallel near-continuous rating of all emotion categories, each movie scene was cut into 3-10-seconds long clips based on the original cut-points preferring local loudness minima in the soundtrack of the movies to avoid splitting the videos in the middle of continuous actions. Where the time between subsequent cuts exceeded the maximum clip duration of 10 seconds, the cut interval was first equally split into the minimum number of <10-second windows. The splitting points were then adjusted to coincide with the closest loudness minima of the soundtrack. The raters evaluated the intensity of each emotion elicited by the clip using a Likert scale from 0 to 4. Finally, we calculated the average intensity of each emotion category for each movie scene by averaging across the scene-wise clips, arranged movie scenes by the most intense emotion categories they elicited, and manually selected the scenes to cover a wide and equal range of different emotion categories (see Supplementary Tables S1 and S2).

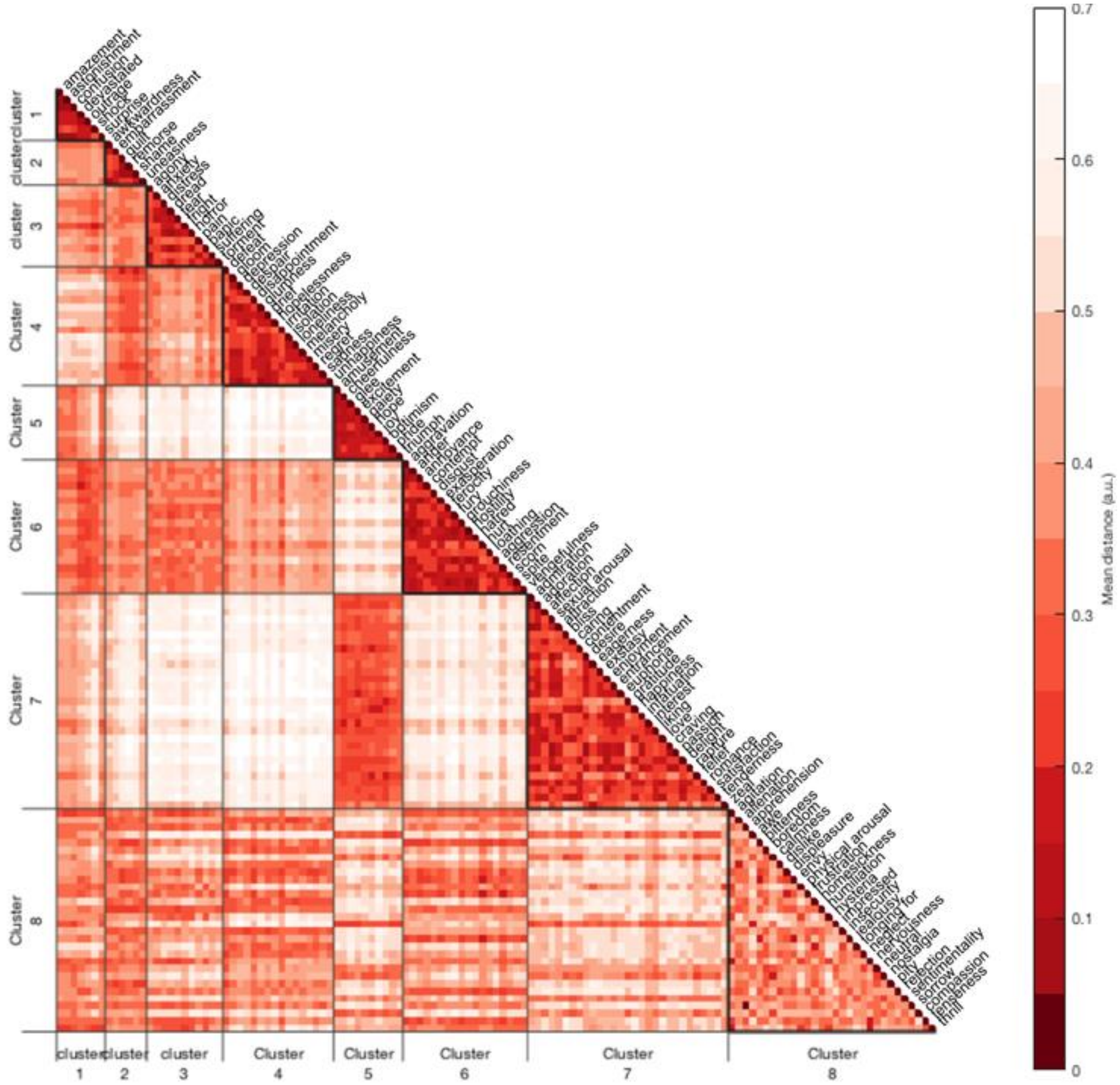

Supplementary Figure S1.

The similarity of emotion categories. To aid the selection of emotion categories that span the entire emotion space, we ran a pilot study (N=25 participants) to collect similarity ratings between our original list of 127 emotion categories. The ratings were collected using an online sorting tool (https://version.aalto.fi/gitlab/eglerean/sensations; initially used in Nummenmaa, L., Hari, R., Hietanen, J. K., & Glerean, E. (2018). Maps of subjective feelings. *Proceedings of the National Academy of Sciences*, *115*(37), 9198-9203). Clusters were identified with density-based clustering (DBSCAN; Ester, M., Kriegel, H. P., Sander, J., & Xu, X. (1996) A density-based algorithm for discovering clusters in large spatial databases with noise. In *kdd* (Vol. 96, No. 34, pp. 226-231)).

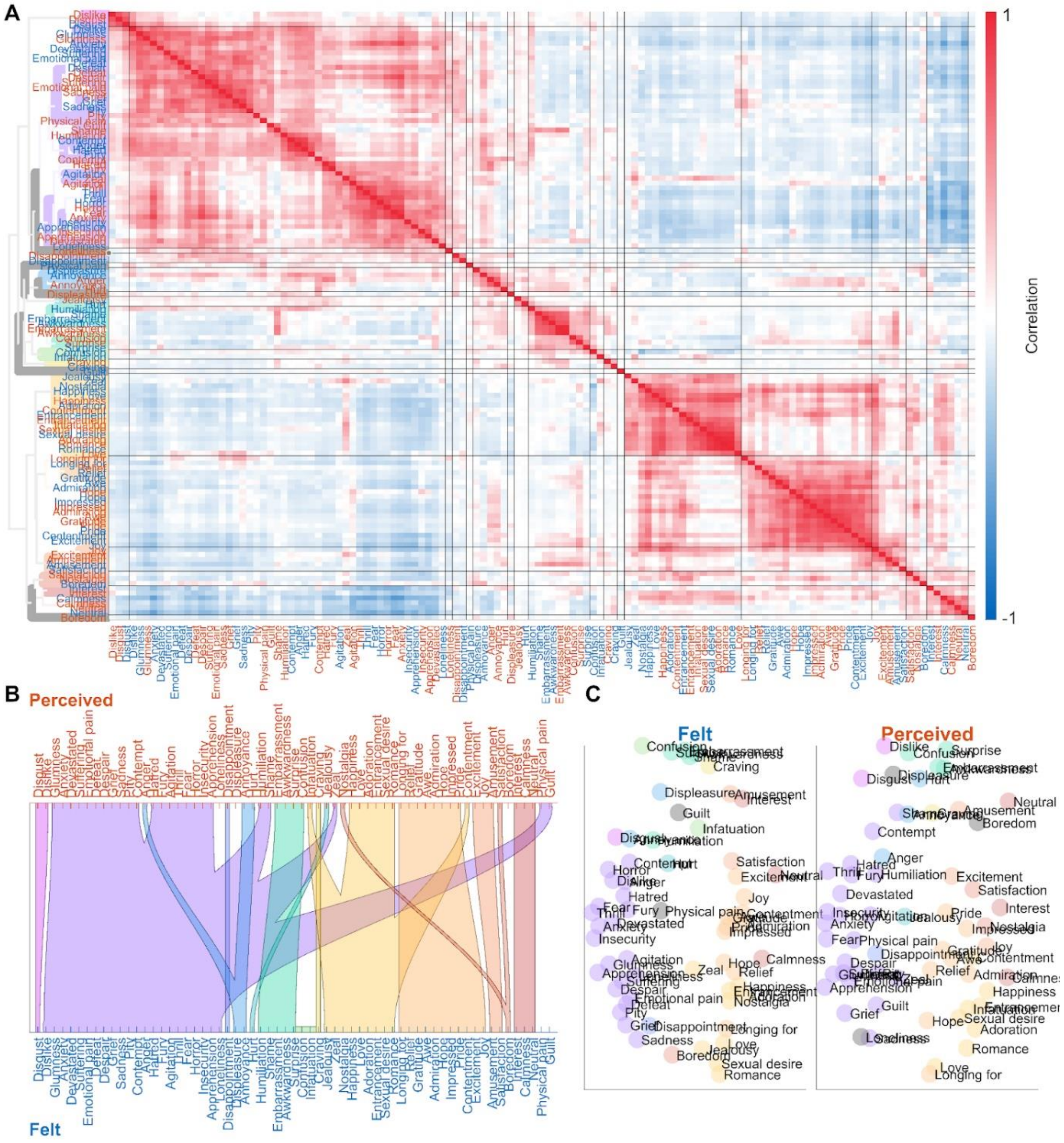

Supplementary Figure S2.

Cluster structure for all emotions based on ratings. A Correlation matrix and dendrogram of ratings over emotion models across all runs and both felt and perceived emotions in the set of reliable emotion ratings. **B** Alluvial diagram shows cluster labels' correspondence between felt and perceived emotions. **C** Multidimensional scaling visualizes the similarity of emotions based on only the ratings of felt emotion (top) and perceived emotions (bottom). The emotions that did not belong to any cluster are shown in gray in the dendrogram and multidimensional scaling plots and are left unconnected in the alluvial diagram.

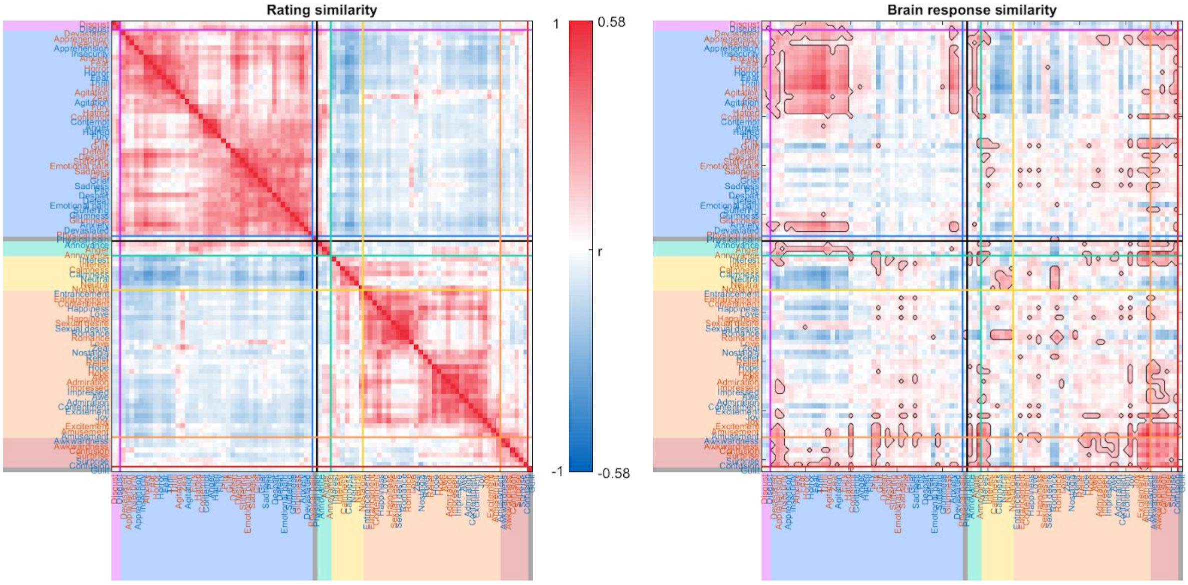

Supplementary Figure S3.

Similarity between emotion categories. The similarity between emotion categories is shown for emotion ratings (left) and cross-validated reliability and spatial correlation of brain responses to the emotions (right). Emotions are organized by the rating clusters in Figure 2. Significant similarity of brain responses compared with randomly sampled data (p<.05, uncorrected) is indicated by black contours.

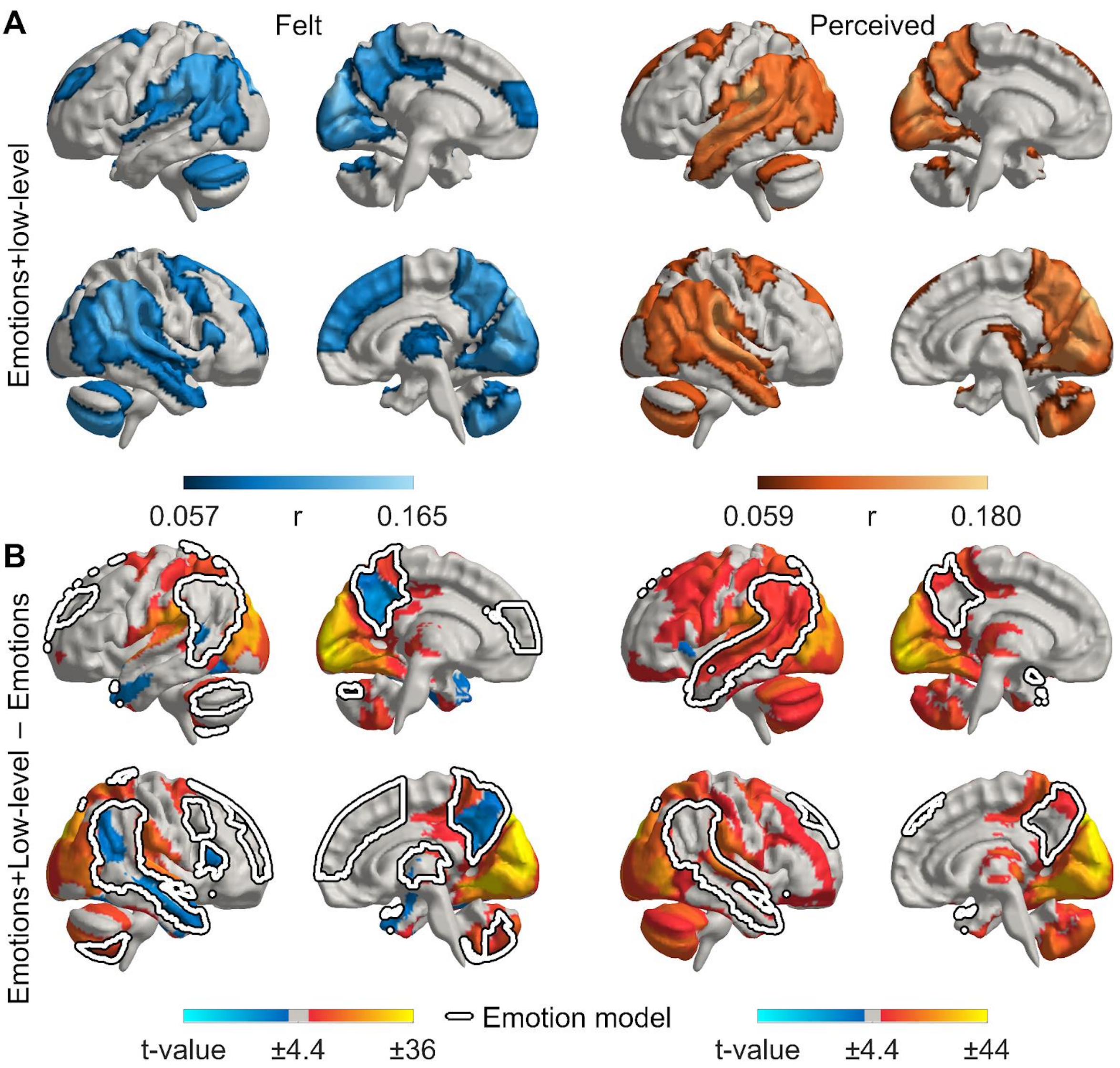

Supplementary Figure S4.

Generalization of emotion models extended with low-level and semantic stimulus features across runs. A Model fits for the extended emotion model. Results are analogous to Fig. 3B, but in addition to emotion models, low-level visual (amount of high spatial frequencies and differential energy between subsequent frames) and auditory (root-mean-squared power) and semantic (panoptic segmentation) features were added into the model. **B** Contrast of model fits for the extended emotion+low-level+semantic models vs. emotion-only models. Hot colors indicate where the low-level and semantic stimulus features improved model fit, cold colors indicate areas where stimulus features decreased the cross-validated model fit, presumably due to overfitting the training data. The contrast is thresholded at $p<.05$, Bonferroni corrected over ROIs and the two emotion models (corrected $p=.05/273/2$). The outlines indicate areas where the between-runs model fit was significant for the emotion models in Fig. 3B.

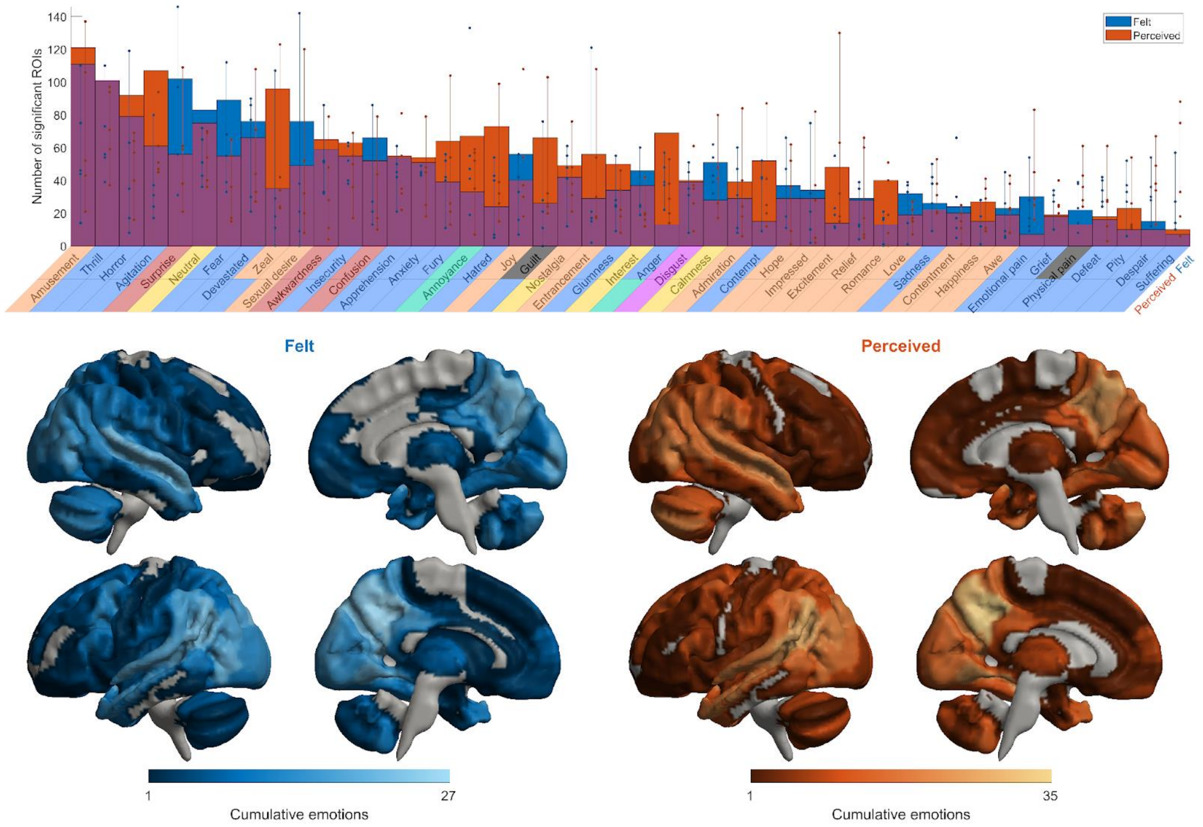

Supplementary Figure S5.

Total brain area activated by individual emotions. Top: Total brain area activated by individual emotions (**top**) colored by clusters derived from spatial clustering of neural responses. **Bottom**: Cumulative maps show the number of emotions whose responses were statistically significant in at least one run. The data are thresholded at two-tailed $p < .05$ (FWER corrected).

Supplementary Table S1.

Region-wise correlations for the cross-validated emotion model fitting.

ROI	Centroid MNI coordinates (mm)				
	X	Y	Z	Felt corr. (mean+-std)	Perceived
SFG_R	9.5	19.3	57.2	0.07+-0.05	
SFG_L	-8.6	52.3	42.4		0.06+-0.05
SFG_R	15.7	51.5	43.1	0.10+-0.04	0.07+-0.04
SFG_R	8.8	41.5	38.1	0.06+-0.05	
SFG_L	-4.6	59.6	18.0	0.06+-0.04	
SFG_R	10.6	61.7	15.7	0.08+-0.05	
MFG_L	-25.0	46.0	33.8	0.06+-0.04	
MFG_R	45.2	14.5	41.5	0.06+-0.05	
MFG_R	30.7	58.6	19.6	0.07+-0.04	
IFG_R	57.2	26.9	14.0	0.09+-0.05	
STG_L	-60.0	-30.0	9.6		0.08+-0.02
STG_R	69.4	-17.1	8.7		0.06+-0.02
STG_R	50.1	15.6	-17.3	0.07+-0.03	0.07+-0.04
STG_L	-52.5	-0.2	-7.9		0.09+-0.03
STG_R	59.4	-9.0	-2.5		0.08+-0.03
MTG_L	-51.6	4.4	-25.3		0.08+-0.04

MTG_R	55.1	8.2	-28.2	0.07+-0.04	0.09+-0.05
MTG_L	-56.7	-54.9	7.0	0.08+-0.04	0.07+-0.03
MTG_R	63.3	-50.9	5.3	0.07+-0.05	
MTG_L	-56.1	-16.6	-7.1		0.09+-0.04
MTG_R	61.1	-13.1	-7.4	0.07+-0.03	0.08+-0.03
pSTS_L	-52.2	-37.2	6.7		0.09+-0.04
pSTS_R	55.7	-34.2	6.1	0.08+-0.03	0.09+-0.04
pSTS_L	-50.1	-47.5	13.4	0.08+-0.03	0.07+-0.03
pSTS_R	59.9	-37.4	15.4	0.11+-0.03	0.06+-0.03
IPL_L	-53.5	-46.7	40.2	0.10+-0.04	0.07+-0.04
IPL_R	60.3	-40.9	41.0	0.11+-0.05	0.07+-0.04
IPL_L	-44.2	-62.5	28.8	0.08+-0.04	0.07+-0.03
IPL_R	56.1	-51.8	27.5	0.12+-0.04	0.08+-0.03
Pcun_L	-2.3	-61.0	54.0	0.10+-0.04	0.10+-0.04
Pcun_R	8.9	-62.5	53.9	0.10+-0.04	0.09+-0.03
Pcun_L	-5.1	-44.7	60.1	0.07+-0.03	
Pcun_R	10.3	-44.5	61.5	0.06+-0.03	
Pcun_L	-3.3	-52.1	36.5	0.08+-0.04	0.09+-0.04
Pcun_R	9.3	-51.3	37.5	0.09+-0.03	0.10+-0.04
Tha_R	9.5	-8.5	8.4	0.06+-0.04	

Tha_R	12.4	-11.3	16.9	0.06+-0.04
Cer_L	-33.7	-66.6	-29.7	0.07+-0.04
Cer_L	-23.8	-73.6	-39.8	0.08+-0.03
Cer_V	1.2	-66.0	-29.1	0.06+-0.03
Cer_V	2.3	-65.1	-35.9	0.06+-0.04
Cer_V	1.9	-61.4	-39.5	0.06+-0.04

Supplementary Table S2.

List of emotion categories. Green = emotion categories chosen based on earlier studies ([1] Cowen & Keltner 2017; [2] Skerry & Saxe 2015; [3] Saarimäki et al. 2018; [4] Adolphs 2002). Yellow = emotion categories added based on pilot similarity ratings (N=25) from the emotion list by Shaver et al. (1987).

Cluster	Emotion word (FIN)	Emotion word (ENG)	Primary reference
1	hämmennys	confusion	1
1	pöyristyneisyys	devastated	2
1	hämmästys	surprise	1-5
2	kiusaantuneisuus	awkwardness	1
2	nolous	embarrassment	2,4-5
2	syyllisyys	guilt	2-5
2	häpeä	shame	2.5
3	ahdistus	anxiety	1.5
3	pelko	fear	1, 3-5
3	kauhu	horror	1, 5
3	fyysinen kipu	physical pain	1
3	henkinen kipu	emotional pain	1
3	kärsimys	suffering	5
4	häviäminen	defeat	5
4	epätoivo	despair	3, 5
4	pettymys	disappointment	2, 5
4	synkkyys	glumness	5
4	murhe	grief	5
4	yksinäisyys	loneliness	2, 5
4	suru	sadness	1, 3-5
5	huvittuneisuus	amusement	1, 5
5	innostus	excitement	1-2, 5
5	toiveikkuus	hope	2, 5
5	ilo	joy	1-2, 5
5	ylpeys	pride	2-5
6	suuttumus	anger	1, 3-5
6	ärtymys	annoyance	2, 5
6	paheksunta	contempt	3-5
6	inho	disgust	1-5
6	raivo	fury	2, 5
6	viha	hostility	5
6	loukkaantuneisuus	hurt	5
7	ihailu	admiration	1, 4
7	ihastus	adoration	1, 5
7	tyytyväisyys	contentment	2, 5
7	halu	sexual desire	1
7	lumoutuneisuus	entrancement	1
7	kiitollisuus	gratitude	2.3
7	onnellisuus	happiness	3-5
7	hullaantuminen	infatuation	4.5
7	kiinnostus	interest	1

Cluster	Emotion word (FIN)	Emotion word (ENG)	Primary reference
7	rakkaus	love	2,4-5
7	himo	craving	1
7	helpotus	relief	1, 5
7	romanttisuus	romance	1
7	tyydytys	satisfaction	1
7	kiihko	zeal	5
8	huolestuneisuus	apprehension	2, 5
8	syvä kunnioitus	awe	1
8	ikävystyminen	boredom	1
8	rauhallisuus	calmness	1
8	vieroksuminen	dislike	5
8	tyytymättömyys	displeasure	5
8	kiihtymys	emotional arousal	5
8	nöyryytys	humiliation	5
8	vaikuttuneisuus	impressed	2
8	epävarmuus	insecurity	5
8	mustasukkaisuus	jealousy	2, 5
8	kaipaus	longing for	3, 5
8	neutraali	neutral	3
8	nostalgia	nostalgia	1, 2
8	sääli	pity	5
8	jännitys	thrill	5

Supplementary Table S3.

Content and duration of the movie clips.

Clip number	Original movie	Duration	Content
3	The dead poet's society	4:14	Todd (Ethan Hawke) commits suicide.
7	Seven	1:43	Crime linked to the sin of sloth. A man apparently dead is discovered lying on a bed, his hands tied. He is extremely skinny, and has been savagely tortured. The word “Sloth” has been written on the walls, and in the room they find pictures depicting different stages of the victim’s progressive demise. Unexpectedly, the man wakes up.
9	E.T.	4:35	E.T. is going to die, surrounded by scientists.
10	Trainspotting	1:37	In an apartment, several persons are sleeping. Then, a women screams. “Sick Boy” (played by Johnny Lee Miller) tries to calm her down. In the meantime, the others wake up. They eventually find out that the woman’s newborn baby is dead. After a long silence, Sick boy asks Mark (Ewan McGregor) to say something. Mark then says he will make a “fix”.
14	When Harry met Sally	2:45	In a very well-known scene, Sally (Meg Ryan) fakes an orgasm in the restaurant, provoking Harry’s (Billy Cristal) embarrassment.
15	Forrest Gump	2:01	The child is introduced to Forrest. The boy goes to sit down in front of the television. Jenny – the mother – tells Forrest that this is his son. Forrest sits down close to the boy. Very moved, Jenny watches the father and the son sitting close to each other.
16	Scream	6:33	A girl answers to the phone. She is asked what her preferred horror movie is. Progressively, she finds out that the person she is speaking to knows her, and has a serious intention to kill her. Afterwards, she sees her boyfriend being killed by this person. Eventually, the killer, wearing a black coat and a grotesque mask, gets into the house and chases her with a knife. At the end, he manages to kill her.
17	Trainspotting	1:02	After a drunken night, Spud (Ewen Bremmer) wakes up in his girlfriend’s bed, and realizes that the sheets are dirty with his excrements. In the following scene, he tries to hide this from the gilfriend’s mother, who wants to take the sheets for the laundry. In the confusion, the mother pulls the sheets away from Spud’s hands, and accidentally splashes the whole family with excrements.
18	The professional	2:44	Léon (Jean Reno) plans the escape of Mathilda (Nathalie Portman). He puts her in the ventilation circuit. She doesn’t want to leave Leon. He promises that they are going to reunite later. They say goodbye to each other, and Mathilda understands that she is never going to see Leon again.
21	Dead man walking	6:40	Execution of Matthew by injection (Sean Penn): He is tied on the execution table, and the scene shows the lethal substance being progressively injected in his veins.
22	The silence of the lambs	3:29	Extraction of a butterfly’s larva from a dead body’s mouth.
23	A fish called Wanda	2:53	Archie (John Cleese) gets undressed, waiting for his girlfriend. Unexpectedly, the owners of the house get into the house and discover him naked.
25	Sleepers	2:20	The guardian brings the children to the cave, so that he can sexually abuse them.
26	When a man loves a woman	1:39	Alice (Meg Ryan) promises Michael (Andy Garcia) to never acting impulsively again, and she promises to stop drinking.
27	Saving private Ryan	5:22	Beginning of the movie. In Omaha Beach, American troops landing in June 1944. A heavy fighting unfolds, where several soldiers are killed amid several horrible scenes.
28	The shining	4:15	Jack (Jack Nicholson) pursues his wife with an axe.
30	In the name of the father	3:30	Violent interrogation of Gerry (Daniel Day-Lewis), when the interrogators threaten to murder his father. He eventually signs a confession forged by the interrogators.
31	Indiana Jones and the last crusade	1:53	Indiana Jones (Harrison Ford) escapes from a catacomb full of rats, in Venice, under a library.
32	Copycat	2:23	Monahan (Holly Hunter) goes to the toilets to looks for the murderer; she gets caught.
**Clip number**	**Original movie**	**Duration**	**Content**
33	Ghost	3:35	Molly (Demi Moore) and Sam (Patrick Swayze) make pottery together, in a very romantic scene.

35	Trainspotting	1:44	Mark (Ewan McGregor) is a drug addict who has not taken heroin from a while and is suffering from withdrawal symptoms. As a consequence, he suffers from a violent diarrhoea. He is then obliged to go to an extremely dirty public restroom. After defecating, he remembers that he had just hidden a newly purchased heroin pill in his anus. He is then forced to search deep through his excrements for the pill.
36	City of Angels	4:15	Maggie (Meg Ryan) dies in Seth's (Nicolas Cage) arms.
38	IT	2:13	A clown hidden in the sewer attracts a boy.
39	The piano	0:43	Stewart (Sam Neil) cuts off Ada's hand (Holly Hunter) with an axe.
43	A perfect world	4:27	Butch (Kevin Costner) is gunned down, at the end of the movie.
46	Child's play II	1:05	Chucky beats Andy's teacher with a ruler.
48	Life is beautiful	3:48	In a prisoner's camp, the father (Roberto Benigni) translates the orders given by the soldier to the prisoners. He is not actually translating, but he is making up a translation that does not scare his son. Specifically, he is trying to make his son believe that all this is a large-scale game.
49	Blue	0:25	A woman goes up on an escalator, carrying a box.
50	Misery	3:31	Annie (Kathy Bates) breaks Paul's legs (James Caan).
51	Leaving Las Vegas	2:30	Sera (Elisabeth Shue) is raped and beaten by 3 young men.
52	Dangerous minds	2:08	The character played by Michelle Pfeiffer tells the class that one of their classmates is dead.
53	Underground	0:58	A man's attempts to have sex with a prostitute are interrupted by the bombing of the city by German planes during WWII.
55	The Blair witch project	3:57	Anxious-provoking scene by the end of the movie: Heather (Heather Donahue), and Mike (Michael Williams) – who is filming Heather - are looking for Joshua (Joshua Leonard) in the woods, at night. They hear screams, apparently form Joshua. They find a house, from where the screams are coming. Mike and Heather go to the second floor. Next, Mike comes back without Heather. Heather's screams can be heard. Eventually, Mike's camera falls on the ground, and keeps filming.
56	Benny & Joone	2:01	Benny (Johnny Depp) plays the fool in a coffee shop.
57	Hellraiser	1:30	On the floor, the size of two stains are growing, and progressively transforming into a monster with a human-like skeleton.
58	The lover	0:43	Marguerite (Jane March) gets into a car, and the car starts to ride. She is dropped of on an animated street. She knocks on a door, and a Chinese man opens and lets her in.
59	The dead poet's society	2:40	By the end of the movie, all the students climb on their desks to manifest their solidarity with Mr. Keating (Robin William), who has just been fired.
61	There is something about Mary	2:55	Ted (Ben Stiller) fights with the dog.
62	Philadelphia	5:28	Andrew (Tom Hanks) and Joe (Denzel Washington) listen to an opera aria on the stereo. Ted describes to Joe the pain and passion felt by the opera character.
64	Blue	0:16	A person passes a piece of aluminium foil through the window of a car.

Due to their large size, the following supplementary materials are shared online with a permanent identifier:

Supplementary Table S4.

Mean felt emotion intensity ratings (range 0-4) for each movie scene. For a downloadable, color-annotated table, see 10.6084/m9.figshare.20418489.

Supplementary Table S5.

Mean perceived emotion intensity ratings (range 0-4) for each movie scene. For a downloadable, color-annotated table, see 10.6084/m9.figshare.20418489.

Supplementary Table S6.

Emotion-specific consistency across runs for each region of interest. For a downloadable table, see 10.6084/m9.figshare.20418489.

Supplementary Table S7.

Mean *t* values for each region of interest ordered by cluster ($p<.05$, Bonferroni corrected over regions and emotions). For a downloadable table, see 10.6084/m9.figshare.20418489.

Task instructions

Original Finnish welcome text

Kiitos osallistumisestasi Emotional Ratings -tutkimukseen! Tämä osa koskee koettuja/havaittuja tunteita.
Tehtäväsi on katsoa videoita, jotka kuvaavat tunteellisia tilanteita.
Pyydämme sinua arvioimaan videoiden herättämiä tunteita 63 eri tunnetta kuvaavalla asteikolla, vastaten kysymykseen:

[Havainto]
Kun katsot tämän videon, mitä tunteita HAVAITSET ELOKUVASSA, toisin sanoen mitä tunteita (kaikki) kohtauksessa esitetyt henkilöt kokevat ja/tai mitä tunteita kohtauksen tunnelmalla pyritään välittämään?
[/Havainto]

[Kokemus]
Kun katsot tämän videon, mitä tunteita SINÄ koet?
[/Kokemus]

Kaikkien videoiden arviointi kestää yhteensä noin 6–8 tuntia ja voit suorittaa arvioinnit oman aikataulusi mukaan.
Kaikkia arviointeja ei tarvitse suorittaa kerralla, eikä se ole myöskään suositeltavaa.
Arviointien teko voi olla vaativaa sekä keskittymisen että omien tunteiden kannalta, joten pyydämme tekemään arviot osissa.
Parhaiden tulosten saavuttamiseksi **suosittelemme, että pidät tauon vähintään tunnin välein, ja pidemmän tauon (vähintään 30min) noin 2–3h tunnin välein.**
Ennen kuin aloitat arvioinnit, varmista, että olet rauhallisessa paikassa ilman häiriötekijöitä (esim. laita puhelin pois päältä, sulje sähköposti- ja pikaviestiohjelmat, varmista että et ole nälkäinen ja olosi on muutenkin mukava).
Luotettavia tuloksia varten on tärkeää, että pystyt keskittymään arviointeihin suhteellisen neuraalilla olotilalla.
Tee olosi rennoksi ja viihtyisäksi ja nauti elokuvista :)
Arviointituloksesi tallentuvat automaattisesti joka kerta, kun painat "Jatka"-painiketta arvioituasi videon kaikilla asteikoilla.
Kukin video on jaettu lyhyisiin leikkeisiin.
Jos joudut keskeyttämään videoleikkeiden arvioinnin kesken videon, suosittelemme, että aloitat arvioinnin uudelleen kyseisen videon alusta.
Tunteet kehittyvät videoiden aikana, ja pitkän tauon jälkeen sinun voi olla vaikea muistaa, mitä videossa on aiemmin tapahtunut.
Jos käytät yhden videoleikkeen arviointiin yli viisi minuuttia, ohjelma muistuttaa sinua tästä ja pyytää palaamaan videon alkuun.
Voit jatkaa istuntoa myöhemmin palaamalla selaimella tähän osoitteeseen (http://bml.becs.aalto.fi/<RESCODE>) ja kirjautumalla sisään käyttäjätunnuksellasi <UID>.

English translation of welcome text

Thank you for your participation in the Emotional Ratings survey! This section is about perceived/perceived emotions.
Your task is to watch videos that describe emotional situations.
We ask you to rate the emotions evoked by the videos on a scale of 63 different emotions, answering a question:
[Perception]
When you watch this video, what emotions do YOU PERCEIVE in the film, i.e. what emotions do (all) the characters in the scene experience and/or what emotions is the mood of the scene trying to convey?
[/Perception]
[Feeling]
When you watch this video, what emotions do YOU experience?
[/Feeling]
All videos take around 6-8 hours to assess in total and you can complete the assessments according to your own schedule.
It is not necessary to complete all the assessments at once, nor is it recommended.
The assessments can be demanding both in terms of concentration and personal emotion, so please do the assessments in parts.
For best results, **we recommend that you take a break at least every hour, and a longer break (at least 30min) about every 2-3hours.**
Before you start the assessments, make sure you are in a quiet place without distractions (e.g. turn off your phone, close email and instant messaging, make sure you are not hungry and are otherwise comfortable).
For reliable results, it is important that you can focus on the assessments in a relatively neural state.
Make yourself relaxed and comfortable and enjoy the movies :)
Your rating results are automatically saved every time you press the "Continue" button after rating the video on all scales.
Each video is divided into short clips.
If you have to stop rating clips in the middle of a video, we recommend that you start rating again from the beginning of that video.
Emotions develop over the course of the videos, and after a long pause you may find it difficult to remember what happened earlier in the video.
If you spend more than five minutes reviewing a single clip, the software will remind you and ask you to go back to the beginning of the video.
You can resume your session later by returning to this address (http://bml.becs.aalto.fi/<RESCODE>) in your browser and logging in with your <UID>.

Translated with DeepL and manually corrected.

Original Finnish instructions to the rating tool

Arviointityökalu on esitetty alla olevassa kuvassa.
Käytä hetki siihen tutustumiseen.
Kun tunteita kuvaavat asteikot ovat tuttuja, arviointisi ovat luotettavampia.
Arvioinnit on tällöin myös helpompi suorittaa.
Pitkien taukojen jälkeen tutustu arviointityökaluun uudelleen.

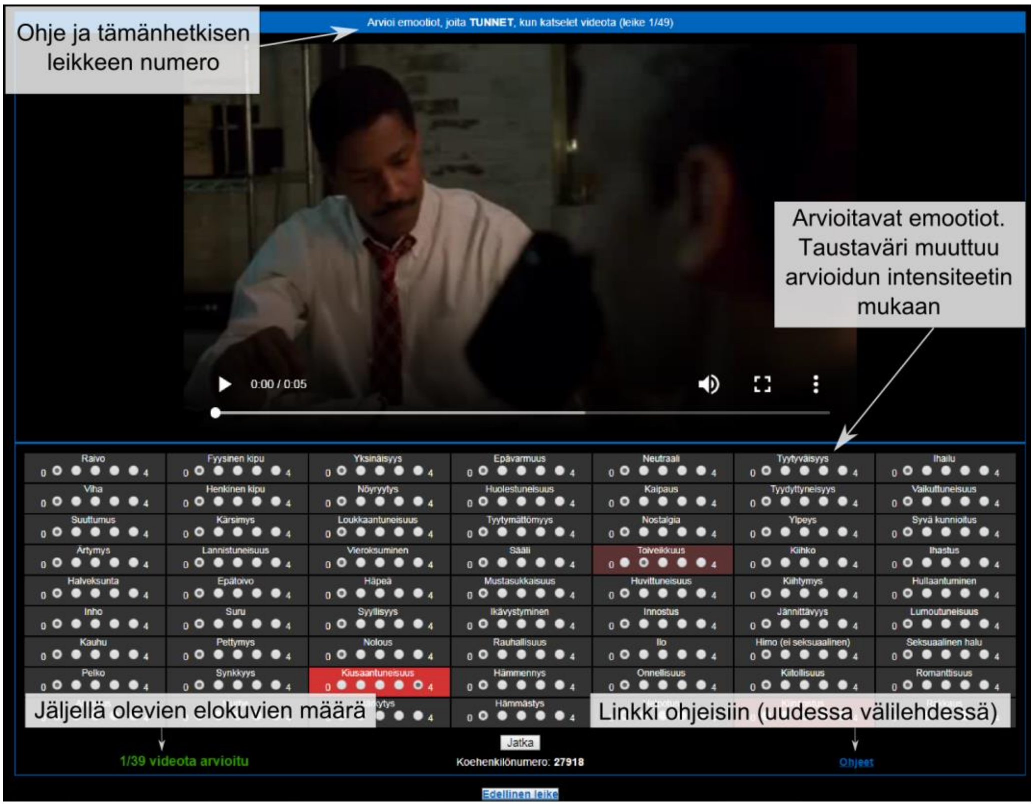

Sinulle esitetään 39 videota.
Videot ovat 16 sekuntia – 7 minuuttia pitkiä.
Videot pysähtyvät noin 3–10 sekunnin välein, jolloin sinua pyydetään suorittamaan arviointi.
Jokaisen lyhyen videoleikkeen jälkeen sinun tulee käydä läpi kaikki asteikot ja arvioida jokainen tunne tehtävän mukaisesti.
Sinun on arvioitava yksittäiseen videoon kuuluvat leikkeet yhden istunnon aikana, mutta voit keskeyttää arvioinnin kunkin kokonaisen videon jälkeen ja palata arvioimaan videoita myöhemmin.
Videoleikkeiden tulisi käynnistyä automaattisesti.
Jos näin ei käy, ota yhteyttä vastuututkijaan (<e-mail>).
Videon yläpuolella näet tehtävänannon ja etenemisesi videon sisällä
(kuinka monta nykyisen videon leikettä on vielä arvioitavana).
Arviointiasteikkojen alapuolella näet kuinka monta videota on vielä arvioitavana, koehenkilönumerosi, *"Jatka"*-painikkeen, linkin ohjeisiin (tämä sivu) sekä *"Edellinen leike"*-painikkeen.
HUOM. Pyydämme, että ette käytä *"Edellinen leike"*-painiketta ellei se ole täysin välttämätöntä.
Tarkoituksena on arvioida videoleikkeet spontaanisti ja jatkuvasti, kun leikkeet nähdään ensimmäistä kertaa.
Käytä *"Edellinen leike"*-painiketta vain hätätapauksissa, esim. jos vahingossa ohitit videoleikkeen arvioimatta tunteita loppuun.

Arviointiprosessi
[Havainto]
Arvioi videoleike jokaisella asteikolla. Tehtävänäsi on arvioida, **kuinka voimaakkaasti ELOKUVAKOHTAUKSESSA ESITETYT HENKILÖT kokevat ja/tai kuinka voimakkaasti kohtauksen tunnelma pyrkii välittämään kyseisiä tunteita** videoleikkeen aikana.

[/Havainto]

[Kokemus]
Arvioi videoleike jokaisella asteikolla. Tehtävänäsi on arvioida, **kuinka voimaakkaasti ITSE TUNNET kyseisiä tunteita** videoleikkeen aikana.
[/Kokemus|

Asteikot kuvaavat tunteen voimakkuutta. Arviot annetaan asteikolla nollasta (0 – tunnetta ei ole lainkaan) neljään (4 – tunnekokemus on erittäin voimakas) vastaavan tunnesanan alla.
Kaikki arvioinnit on asetettu arvoon nolla jokaisen videon ensimmäisen videoleikkeen alussa.
Muutettuasi arvioita nykyiset arviosi kuitenkin ladataan automaattisesti saman videon seuraavan leikkeen alkaessa.
Päivitä arviosi jokaisella asteikolla uuden videoleikkeen katsomisen jälkeen, jos tunne on muuttunut edellisestä leikkeestä.
Halutessasi voit katsoa lyhyen videoleikkeen useamman kerran.
Jos haluat pitää tauon, pidä tauko videoiden välissä, älä saman videon videoleikkeiden välissä.
Tietotekniset vaatimukset
Käytä **koko näytön tilaa (koko näytön tilaan ja siitä pois pääsee useimmissa selaimissa F11-näppäimellä).**
Näytön koon tulee olla vähintään 17 tuumaa ja resoluution 1600x900.
Arvionti tulee tehdä korvakuulokkeita käyttäen,
koska osa äänistä saattaa olla hiljaisia, mutta arvioinnin kannalta tärkeitä (esim. kuiskaus).
Varmistathan että kuulokkeet ovat mukavat, eivätkä paina tai muuten häirite sinua arvioinnin aikana.

English translation of instructions

The assessment tool is shown in the figure below.
Please take a moment to familiarise yourself with it.
When you are familiar with the emotional scales, your assessments will be more reliable.
It is also easier to complete the assessments.
After a long break, familiarise yourself with the assessment tool again.

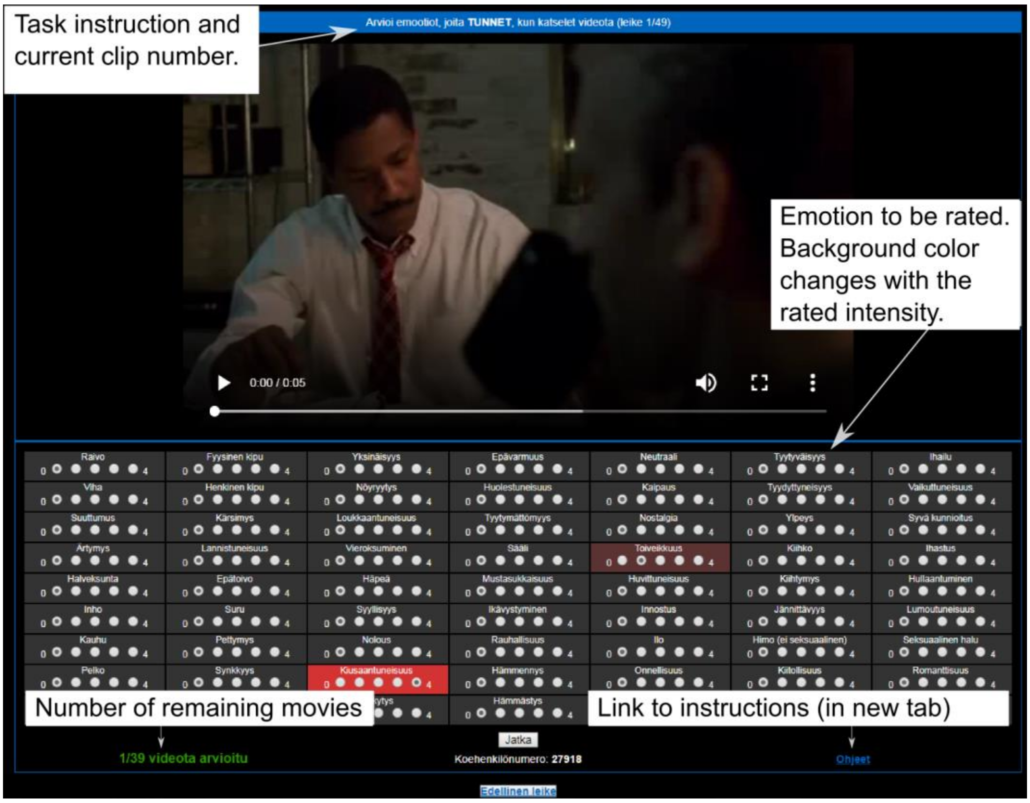

You will be shown 39 videos.
The videos are between 16 seconds and 7 minutes long.
The videos stop every 3-10 seconds or so, at which point you will be asked to complete an assessment.
After each short video clip, you will be asked to go through all the scales and rate each emotion according to the task.
You must assess the clips in a single video in one session, but you can pause the assessment after each complete video and return to assess the videos later.
Video clips should start automatically.
If this is not the case, please contact the researcher in charge (<e-mail>).
Above the video you can see the assignment and your progress within the video
(how many clips of the current video are still to be assessed).
Below the grading scales, you will see how many videos are still to be graded, your subject number, the "Continue" button, a link to the instructions (this page) and the "Previous clip" button.
NOTE: Please do not use the "Previous clip" button unless absolutely necessary.
The purpose is to evaluate video clips spontaneously and continuously when the clips are viewed for the first time.
Use the "Previous Clip" button only in emergencies, e.g. if you accidentally skip a clip without evaluating the emotion until the end.

Rating process
[Perception]
Rate the video clip on each scale. Your task is to assess **how strongly the PEOPLE in the film clip experience and/or how strongly the mood of the scene tends to convey those emotions** during the clip.

[/Perception]

[Feeling]
Please rate the clip on each scale. Your task is to rate **how strongly YOU FEEL** those emotions during the clip.
[/Feeling]
The scales describe the intensity of the emotion. Ratings are given on a scale from zero (0 - no emotion at all) to four (4 - very strong) under the corresponding emotion word.
All ratings are set to zero at the beginning of the first clip of each video.
However, if you change the ratings, your current ratings are automatically loaded at the start of the next clip of the same video.
Update your ratings on each scale after watching a new clip if the emotion has changed since the previous clip.
If you wish, you can watch a short clip multiple times.
If you want to take a break, pause between videos, not between clips of the same video.

Technical requirements
Use full-screen mode (full-screen mode can be accessed and exited with the F11 key in most browsers).
Screen size must be at least 17 inches and resolution 1600x900.
The assessment should be done using earphones,
as some sounds may be quiet but important for the assessment (e.g. whispering).
Please ensure that the headphones are comfortable and do not cause pressure or otherwise disturb you during the assessment.

Translated with DeepL and manually corrected.
